# Preventability of early vs. late readmissions in an academic medical center

**DOI:** 10.1371/journal.pone.0178718

**Published:** 2017-06-16

**Authors:** Kelly L. Graham, Ogechi Dike, Lauren Doctoroff, Marisa Jupiter, Anita Vanka, Roger B. Davis, Edward R. Marcantonio

**Affiliations:** 1 Department of Medicine, Beth Israel Deaconess Medical Center, Harvard Medical School, Boston, Massachusetts, United States of America; 2 Department of Internal Medicine, University of Texas Southwestern Medical Center, Dallas, Texas, United States of America; Stanford University School of Medicine, UNITED STATES

## Abstract

**Background:**

It is unclear if the 30-day unplanned hospital readmission rate is a plausible accountability metric.

**Objective:**

Compare preventability of hospital readmissions, between an early period [0–7 days post-discharge] and a late period [8–30 days post-discharge]. Compare causes of readmission, and frequency of markers of clinical instability 24h prior to discharge between early and late readmissions.

**Design, setting, patients:**

120 patient readmissions in an academic medical center between 1/1/2009-12/31/2010

**Measures:**

Sum-score based on a standard algorithm that assesses preventability of each readmission based on blinded hospitalist review; average causation score for seven types of adverse events; rates of markers of clinical instability within 24h prior to discharge.

**Results:**

Readmissions were significantly more preventable in the early compared to the late period [median preventability sum score 8.5 vs. 8.0, p = 0.03]. There were significantly more management errors as causative events for the readmission in the early compared to the late period [mean causation score [scale 1–6, 6 most causal] 2.0 vs. 1.5, p = 0.04], and these errors were significantly more preventable in the early compared to the late period [mean preventability score 1.9 vs 1.5, p = 0.03]. Patients readmitted in the early period were significantly more likely to have mental status changes documented 24h prior to hospital discharge than patients readmitted in the late period [12% vs. 0%, p = 0.01].

**Conclusions:**

Readmissions occurring in the early period were significantly more preventable. Early readmissions were associated with more management errors, and mental status changes 24h prior to discharge. Seven-day readmissions may be a better accountability measure.

## Introduction

Hospital readmissions are a common and costly issue, affecting one in five hospitalized Medicare beneficiaries annually [1]. Consequently, risk-stratified 30-day readmission rates have become an accountability measure intended to penalize hospitals with excessive 30-day readmissions [2]. The timing used for this metric has fallen under question [3,4,5] and the metric itself does not consistently correlate with adherence to quality indicators [6,7] or low inpatient mortality rates, [8,9,10]; rather it seems more closely associated with idiosyncratic factors such as hospital volume [7,11]. On a patient level, 30-day readmissions disproportionately affect patients with higher chronic illness burden [12–14], and lower socioeconomic status [15–20], rather than representing factors directly related to the index hospitalization, such as physician cognitive errors or problematic systems of care. Hospitals caring for the most socially and medically vulnerable patient populations face the highest penalties as a result [21]; leading to increasing concerns that readmission penalties may exacerbate healthcare disparities. A better metric would more closely represent preventable readmissions, a construct that is difficult to measure, but for which hospitals and their clinical teams are directly accountable.

The increasing literature on preventability of 30-day readmissions suggests that preventable readmissions are rare events, between 8–20% [22–24], and as low as 1% for the most vulnerable patients facing refractory malignancies [25]. Preventability also appears to decrease within the 30-day window [26, 27], leading experts to propose that 3 or 7-day readmissions may be a more appropriate accountability measure for hospitals [3–5]. Prior work by our group found that the 30 days following hospital discharge are not homogenous, with readmissions occurring within 7 days post-discharge being more related to factors associated with the index hospitalization than those occurring during days 8–30, suggesting that this early period may better represent a window of preventability [12]. Moreover, there are no studies directly comparing preventability in an early vs. late period.

The aim of our current study is to advance what was learned from our prior work by directly measuring preventability within an early and late period during the 30 days following hospital discharge using a standard algorithm and blinded hospitalist physician review [27]. We hypothesize that there will be significantly higher rates of preventability in the early than in the late period. Our secondary aims include a comparison of causal factors contributing to readmissions, and rates of markers of clinical instability within the 24 hours preceding hospital discharge between early and late readmission. We hypothesize that early readmissions are more likely caused by factors related to the index hospitalization, such as diagnostic and therapeutic errors of cognition, and discharging clinically unstable patients.

## Methods

### Setting and study population

We selected admission-readmission dyads for chart review from a previous retrospective single center cohort study consisting of 7,974 unique patient admissions from which 1,571 experienced an unplanned 30 day readmission (19.7% readmission rate) at a large urban teaching hospital, which included all medicine admissions (including observation status admissions) from the hospital’s primary care network from 1/1/2009-12/31/2010. We selected a timeframe prior to the institution of a readmissions reduction program in order to explore the relationships under study. Admissions followed by elective readmissions, psychiatry transfers, and long-term acute care facility transfers, were excluded because the nature of their healthcare utilization was felt to be qualitatively different from patients discharged in stable condition. Our sampling strategy ensured that 50% of our sample was derived from the pool of patients that were readmitted within 7 days of hospital discharge (early readmissions), and 50% of our sample was derived from the pool of patients that were readmitted between days 8–30 after hospital discharge (late readmissions). Therefore, our population contained a random sample of 60 unique early admission readmission dyads, and 60 unique late admission-readmission dyads.

### Data sources

We derived the cohort and admission characteristics using the hospital’s clinical and administrative data repositories; the professional fee billing repository; electronic provider order entry data; and electronic nursing assessment tool, which nurses use to collect clinical and socioeconomic data on patients at the time of admission. All of the aforementioned data are prospectively collected as part of regular hospital operations. We abstracted our case summaries and clinical markers of instability from direct chart review for both the index admission and the readmission. The hospital’s Institutional Review Board approved the study with a waiver of informed consent.

### Data extraction

#### Case summaries

Two authors (KG and OD) created a standardized data extraction tool to develop a case summary that summarized the index admission and readmission for the hospitalist reviewers. The extraction process did not include any potential references to readmission timing in the case summaries, so that case reviewers could be blinded to the independent variable. The 2 abstractors pilot tested the data extraction tool among themselves, and the physician reviewers (AV, MJ, LD) to ensure completeness and usability. Once the tool was optimized, it was used to extract all 120 case summaries from the full online medical record.

#### Markers of clinical instability

In parallel with creating the case summaries, two authors (KG and OD) reviewed the full medical record from the index admission to determine whether any of the clinical markers of instability were present in the 24 hours prior to discharge from the index admission similar to those used in prior studies [28,29], Markers of clinical instability included documented falls, changes in mental status, lab or vital sign abnormalities indicating clinical instability, patient declining rehabilitation after hospitalization despite recommendation, or changes in the route of a medication from intravenous to oral.

### Physician review and adjudication

Two hospitalist physicians (AV and MJ) blinded to the readmission timing reviewed each of the 120 case summaries and used a standard preventability algorithm [22, 27, 30] to determine the binary outcome of preventability. This approach requires reviewers to assess both causality (see S1 Fig) and preventability on a 1–6 Likert scale, and classifies a preventable readmission as one with greater than or equal to 4/6 on both scales (See S2 Fig). Where there was disagreement on the binary outcome, a third blinded hospitalist adjudicated the case.

### Measures

#### Independent variable

We defined early readmissions as readmissions that occur within 7 days of discharge from the index hospitalization, and late readmissions as readmissions that occur between days 8 and 30 after discharge from the index hospitalization

#### Primary outcome

**Preventability of the readmission:** The primary outcome was the average preventability sum score for each case. We first added the preventability scores (using the 1–6 scale) across each of the causation categories for each reviewer. We then took the average of these sum scores from the 2 hospitalist reviewers (3 if adjudication was necessary) to create an average preventability sum score for each of the 120 cases. We also included the binary measure of preventability using the standard approach in the field (22, 27, 30). In this approach, to be called “preventable”, the readmission must have both a causation and preventability score greater than or equal to 4 for one or more of the causation categories, and consensus from the two reviewers. If the two reviewers disagreed on this preventability determination, the case was reviewed by a third expert blinded to the 2 previous reviews. The final preventability determination was based on this third reviewer.

#### Secondary outcomes

**Causation of the readmission**: During case review, the hospitalist reviewers used the same six-point ordinal scale to score the causation of each of the potential causal events (in addition to preventability), see S1 Fig and S2 Fig. We calculated a mean causation score for each of the seven causation domains (see S1 Fig) from each of the two reviewers (or three if adjudication was necessary). We used identical methods to calculate a mean preventability score for each of the seven causation domains.

**Markers of clinical instability 24 hours prior to discharge:** We calculated frequencies of six markers of clinical instability 24h prior to discharge from the index admission documented in the online medical record. These are listed above (see the section under Data Extraction).

### Analysis

Because the average preventability sum scores were not normally distributed, we used a Wilcoxon rank sum test to compare the medians of the average preventability sum scores between the early vs. late readmissions.

For similar reasons, we used a Wilcoxon rank sum test to compare the medians of the average causation scores and average preventability of causation scores for each of the seven factors, between the early vs. late readmissions.

Finally, we used a Fisher’s exact test to compare rates of the presence of each of the markers of clinical instability between the early and late readmissions.

Data management and analysis was performed using SAS version 9.3 (Cary, NC).

All relevant data are within the paper and its Supporting Information files. (S1 File and S2 File)

## Results

### Cohort characteristics

The cohort consisted of a random sample of 120 unique patient admission-readmission dyads, 60 from each of the readmission periods derived from a larger cohort study. Patients readmitted within the early period had longer lengths of stay (median length of stay 3.9 vs. 3.1 days, p = 0.04), had more consultants involved in their care (mean number of consultants 1.1 vs 0.6, p = 0.02), and were more likely to have experienced a rapid response secondary to clinical decompensation (15% vs. 3%, p = 0.02) than those readmitted in the late period (Table 1).

**Table 1:** Initial hospitalization and patient characteristics by readmission status.

Variable	Early Readmissions (0–7 days) (n = 60, 50%)	Late Readmissions (8–30 days) (n = 60, 50%)	p value^3^
Length of stay (index admission, days), median, (Q1-Q3)	3.9 (2.4–6.8)	3.1 (1.9–4.4)	**0.04**
Proportion of the hospitalization that a single physician attended on the patient, mean (SD)	0.7 (0.3)	0.7 (0.2)	0.5
Proportion of the hospitalization that the discharging physician attended on the patient, mean, (SD)	0.6 (0.3)	0.7 (0.3)	0.1
Number of consultants, mean, (SD)	1.1 (1.4)	0.6 (1.0)	**0.02**
Discharge Timing (%)			
0800–1259	5	10	0.14
1300–1759	80	63	
1800–0759	15	27	
Disposition on discharge (%)			
Home, no services	50	52	0.9
Home, with services	32	32	
Hospice	0	0	
Skilled nursing facility	18	15	
Rehabilitation hospital	0	2	
Other	0	0	
Number of nursing units, mean (SD)	1.5 (1.0)	1.4 (2.9)	0.7
Age decades (%)			
Less than 50 years	18	18	0.6
50–59 years	18	10	
60–69 years	17	17	
70–79 years	17	27	
Greater than/equal to 80 years	30	28	
Charlson Comorbidity Index, mean (SD)	2.7 (2.4)	2.6 (2.4)	0.8
Chronic Illness Burden (%)			
On medication indicating organ failure	20	20	1.0
On hemodialysis	0	2	0.3
Prior Use of Healthcare, median (Q1 -Q3)			
#Admissions in the last 12m	0.5 (0–1.0)	1.0 (0–1.5)	0.5
Rapid response during admission (%)	15	3	0.02
Intensive care unit stay during admission (%)	20	12	0.2
Laboratory- based acute physiology score, mean (SD)^1^	18.1 (16.0)	23.5 (14.5)	0.06
Gender (% Female)	53	57	0.7
Race (%)			
White	73	65	0.5
Black	20	30	
Hispanic	2	3	
Other	5	2	
Marital Status (%)			
Married/Partnered	43	37	0.4
Single	25	37	
Widowed	18	20	
Divorced/Separated	13	7	
Education Level (%)			
Highschool/GED	23	43	0.6
>Highschool	53	47	
<Highschool	20	10	
Health literacy (%)			
Barriers to learning	48	40	0.4
English speaking	92	90	0.8
Insurance Payer (%)			
Private/Commercial/Med Adv/Liab^2^	48	35	0.02
Medicare/Medicaid	45	62	
Free care/Self pay	0	3	
Other	0	0	

^1^Based on Escobar et. al’s risk prediction model for inpatient and 30 day mortality using laboratory data prior to, or very early after admission.

^2^ Forms of comprehensive insurance coverage, including private, Medicare advantage, and liability insurance

^3^Using a t test for continuous variables, a Wilcoxon rank sum test for ordinal categorical variables, and a Fishers exact test for nominal categorical variables.

### Preventability of early and late readmissions

Based on expert clinician adjudicated review, we found a significant difference in the average sum preventability scores of early vs. late readmissions (median sum score 8.5 vs. 8.0, p = 0.03) (Table 2). Overall, readmissions within 30 days were scored as preventable 15% of the time, with 20% of early readmissions, and 10% of late readmissions meeting the binary definition of preventability (Table 2). Finally, our sensitivity analysis restricting the preventability sum score to cases meeting the binary definition of preventability did not appreciably alter the results.

**Table 2:** Blinded physician-adjudicated readmission preventability score by readmission status.

	Early Readmissions (0–7 days) (n = 60)	Late Readmissions (8–30 days) (n = 60)	p^4^
Preventable [%]^1^	20	10	0.13
Median [Q1-Q3] Preventability sum score^2^	8.5 [7.5–10.4]	8.0 [7.0–9.0]	**0.03**
Median [Q1-Q3] Preventability sum score^3^	8.5 [7.5–10.4]	7.5 [7.0–8.5]	**0.02**

^1^Defined as a preventability score of > = 4 on a 6 point ordinal scale

^2^A sum score was created for preventability rankings by using the sum of the preventability scores from each of the physician reviewers, and taking the average of the 2 [or 3 if adjudication was necessary]. The median and quartiles are presented.

^3^As a sensitivity analysis, we also conducted the primary analysis using a late cut-off of 15–30 days to ensure validity of our findings

^4^Using a Wilcoxon rank sum test comparing median preventability sum scores

### Factors contributing to early vs. late readmissions

Physician reviewers found significantly more management errors in the early period than in the late period (mean causation score 2.0 vs. 1.5, p = 0.02) (Table 3). Similarly, physician reviewers found that the preventability of management errors was significantly higher in the early period compared to the late period (mean preventability of causation score 1.9 vs 1.5, p = 0.03) (Table 3). There was no difference in the rates or preventability of other types of errors (Table 3).

**Table 3:** Physician reviewers’ causation and preventability ratings of factors contributing to readmission.

Factors Contributing to Readmission^1^	Mean Causation Score [SD]^2^ Early Readmissions [0–7 days] n = 60	Mean Causation Score [SD]^2^ Late Readmissions [8–30 days] n = 60	p^3^	Mean Preventability Score [SD]^4^ Early Readmissions [0–7 days] n = 60	Mean Preventability Score [SD]^4^ Late Readmissions [8–30 days] n = 60	p^5^
Medication. related event	1.8 [1.4]	1.6 [1.3]	0.9	1.4 [1.0]	1.2 [0.5]	0.8
Procedure related event	1.1 [0.5]	1.1 0.5]	1.0	1.0 [0.3]	1.0 [0.1]	0.7
Nosocomial infection	1.2 [0.7]	1.2 [0.5]	0.6	1.0 [0.15]	1.0 [0.2]	0.7
Diagnostic error	1.4 [0.9]	1.3 [0.7]	0.7	1.3 [0.7]	1.2 [0.6]	0.9
Management error	2.0 [1.2]	1.5 [0.8]	**0.02**	1.9 [1.2]	1.5 [0.8]	**0.03**
Systems error	1.4 [0.6]	1.3 [0.5]	0.1	1.3 [0.5]	1.2 [0.3]	0.1
Surgical complication	1.1 [0.4]	1.1 [0.3]	0.8	1.0 [0.1]	1.0 [0.2]	0.6

^1^Physician reviewers used a list of 7 potential factors that led to the readmission to determine both causation of the readmission, and preventability [See S2 Fig]

^2^We created a mean causation score for each of the listed factors based on our reviewers ratings, presented by readmission status

^3^Using a Wilcoxon rank sum test comparing median causation sum scores between the early and late readmissions

^4^We created a mean preventability score for each of the listed factors based on our reviewers ratings, presented by readmission status

^5^Using a Wilcoxon rank sum test comparing median causation sum scores

### Markers of clinical instability 24h prior to hospital discharge

We found significantly more documented episodes of an acute mental status change on the day of discharge from the index admission for early readmissions compared to late readmissions (12% vs. 0%, p = 0.01) (Table 4). There were no other significant differences in markers of clinical instability on the day of discharge between the early and late readmissions.

**Table 4:** Frequency of markers of clinical instability 24 hours prior to hospital discharge by readmission status.

	Early Readmissions [0–7 days] [n = 60]	Late Readmissions [8–30 days] [n = 60]	p-value^1^
Any marker [%]	55	48	0.6
Falls [%]^2^	0	0	na
Mental Status Changes [%]^2^	12	0	**0.01**
Ordering of a lab indicating clinical instability [%]^3^	8	17	0.3
Vital sign abnormality indicating clinical instability [%]^4^	37	30	0.6
Patient declines recommended acute inpatient rehabilitation post-discharge [%]	0	0	na
Change in medication route from IV to oral [%]^5^	20	22	1.0

^1^Using a Fishers exact test comparing frequencies of the presence of each marker

^2^Documented in the MD or nursing progress note

^3^Including arterial blood gas, lactate, or cardiac enzymes

^4^Including systolic blood pressure >180 or <90, diastolic blood pressure >100 or <50, temperature >100.4, pain score >5

^5^For antibiotics, steroids, diuretics, and narcotic pain medications

## Discussion

In this study using blinded hospitalist-adjudicated reviews of detailed medical record abstractions, we found a significant difference in preventability scores between an early and late period within the 30 days following hospital discharge. We also found higher scores for preventable management errors in the early vs. late periods, and a higher rate of acute mental status changes 24h prior to the index hospital discharge in the early vs. late readmissions. These results suggest that readmissions occurring within the first week following hospital discharge may be more preventable than those occurring later in the 30-day window following hospital discharge.

Our findings support our hypothesis that rates of preventability are higher in the early than in the late period. These findings are also consistent with our prior work, which showed that factors related to the index hospitalization, such as acute illness burden and discharge timing were more closely associated with early readmissions than late readmissions, which were more closely associated with chronic illness burden and social factors, such as health literacy and insurance payer [12]. We conducted a sensitivity analysis using a more disparate cut-off than the primary analysis (comparing days 1–7 to days 15–30), and noted an increase in the effect size, further supporting the validity of our findings (Table 2). We were able to address our hypothesis more directly in the current study with a specific measure of preventability, rather than simply relying on a change in correlations within the 30-day window. Notably, we purposefully addressed our hypothesis during 2009–2010, prior to the implementation of readmission reduction programs at our institution, allowing us to evaluate the validity of the measure prior to active mitigation efforts.

We also found that the overall rate of preventability in the 30 days following hospital discharge was 15%. These findings are also consistent with studies that used similar methods in Canada, and found a rate of preventability of 16% [22], as well as other studies in the US literature, where rates vary from 8–21% [23,24]. This consistency suggests that our data is valid despite our small sample size, and that these figures are reliable. Our study is the first of its kind to take this question one step further and divide the 30 days into an early and late period to evaluate the effect of readmission timing on preventability. A recently published multi-center study in the US using the same preventability algorithm found a higher rate of preventability of 30-day readmissions of 29.6% [30]. This study differed in an important way from our study- aside from a different approach to physician review and adjudication, the reviewers used a standard of an ideal health system to judge preventability, where our study considered the limitations of the health care system in its current state. Such an approach would naturally produce the observed difference in rates of preventability, specifically when dealing with faulty systems of care beyond the control of the hospital.

In an attempt to understand more about what factors are contributing to early readmissions, we took full advantage of the level of clinical detail from chart review, and conducted two secondary analyses. The first compared ratings of causation and preventability for seven potential factors (See S1 Fig) that contribute to readmissions, between early and late readmissions. We found that management errors (defined as an instance when a provider orders the incorrect therapy, does not monitor the effects of a therapy correctly, or fails to initiate the indicated therapy), were significantly more common in the early period than in the late period, and had significantly higher preventability scores (Table 3). Management errors represent events that clinical teams operating within hospitals are directly accountable, though not feasible to measure on a large scale. These findings are consistent with prior work [30], where issues related to medication safety and therapeutic problems were found to be contributors to preventability in the 30-day window.

We also took advantage of the granularity of chart review to directly evaluate for markers of clinical instability on the day of discharge. We reviewed the physician and nursing notes 24h prior to discharge for any documentation of markers of clinical instability within the 24 hours prior to hospital discharge. We found significantly more documented evidence of acute mental status changes on the day of discharge for early readmissions compared to late readmissions (Table 4), which has been demonstrated in other studies as contributors to readmissions [28,29]. Premature discharge of acutely confused patients also represent preventable adverse events for which the clinical teams operating within the hospital are accountable, as they are likely a result of physician cognitive errors or faulty communication among team members. It is important to note that for the analysis of clinical markers of instability the physicians collecting the data for analysis (KG and OD) were not blinded to the timing of the readmission. We attempted to mitigate any potential bias by using strict clinical criteria for these outcomes, rather than our judgment. However, these results should be interpreted with this limitation in mind.

Notably, the patients in the early readmission cohort had longer lengths of stay, more consultants, and more frequent rapid responses to clinical instability. This is consistent with our prior work [12], and indicates that patients in the early cohort have higher acute illness burden than those in the late readmission cohort. This is also consistent with our overall conceptual model, which proposes that acute illness burden is more actionable by the admitting hospital than factors related to ambulatory care sensitive conditions and social determinants of health, and as such, would be more common in the early cohort.

Our study has some limitations. It is a single center study, limiting the generalizability of our findings nationally. Our sample size is small, with only 120 admission/readmission dyads available for analysis. However, each case summary was extracted by a physician reviewer, and all cases were reviewed and scored for all outcomes by hospitalist physicians, a level of expertise and detail that is difficult to find in the literature. Moreover, our results are consistent with our prior work [12] and the current literature [23,24], which suggests that our findings provide a valid proof of principle that can be confirmed in future studies. Because of our small sample size, we were underpowered to compare rates of preventability using a dichotomous measure. Fortunately, given the level of data collection for each case summary, we were able to create an average sum score for each case, which allowed us to take full advantage of the data and perform a statistical analysis that was better powered to measure the difference in preventability. Given the limitations in sample size, our findings must be viewed as preliminary work, which should be validated in a larger population.

Our findings have several potential implications for further study. Experts have questioned the validity of the 30-day readmission metric as adequately capturing accountability on the part of the discharging hospital, and some have recommended a shorter window to hold hospitals accountable financially or weighing penalties based on the timing of admissions [3,4,5]. Our study supports these recommendations, suggesting that readmissions may be more likely to be preventable and related to physician cognitive errors and systems errors within the seven days following discharge than within 30 days post-discharge. It also identifies reducing discharge of patients with acute confusion as a quality improvement target.

In summary, we found that readmissions within an early period of seven days post-discharge were more likely to be preventable based on a blinded and adjudicated hospitalist physician review than readmissions occurring within a late period of 8–30 days post-discharge. Early readmissions are more likely to be caused by factors by which the clinical teams operating within the discharging hospital are directly accountable, such as management errors and premature discharge of clinically unstable patients, than late readmissions. These findings support our prior work, and continue to suggest that the 30 days following hospital discharge are a heterogeneous timeframe with respect to accountability.

## Supporting information

S1 Fig Types of events contributing to readmissions, and their definitions [22]. (PDF)

S2 Fig Sample algorithm used to assess causality and preventability of readmissions. (PDF)

S1 File Dataset used for the primary analysis of early vs. late readmissions. (XLSX)

S2 File Dataset used for the sensitivity analysis of preventability of readmissions occurring between days 1–7 and 14–30. (XLSX)
