# Supplementary material for: Preventability of early vs. late readmissions in an academic medical center
Source: PLoS One. 2017 Jun 16;12(6):e0178718. doi: 10.1371/journal.pone.0178718 (PMC5473551; doi:10.1371/journal.pone.0178718)
Supplement: S1 Fig — (PDF) [file pone.0178718.s001.pdf]

S1Fig

Event	Definition
Medication-related event	Patient experiences symptoms as a result of a medication administered during the index admission
Procedure-related event	Patients experience symptoms as a result of a procedure during the index admission.
Nosocomial Infection	Patient acquires an infection during the process of hospital care.
Diagnostic Error	When a provider does not follow an appropriate diagnostic algorithm or does not act on the findings of a diagnostic test.
Management Error	When providers order a contra-indicated therapy, do not monitor the effects of therapies correctly, or do not initiate an indicated therapy.
System Error	When a faulty health care system design leads to inefficiency in care or patient harm.
Surgical Complication	Patients experience symptoms as a result of a surgical procedure.
