# Supplementary material for: Preventability of early vs. late readmissions in an academic medical center
Source: PLoS One. 2017 Jun 16;12(6):e0178718. doi: 10.1371/journal.pone.0178718 (PMC5473551; doi:10.1371/journal.pone.0178718)
Supplement: S2 Fig — (PDF) [file pone.0178718.s002.pdf]

S2 Fig:

Scale: Below is the scale you will use for adverse events

	**Causation**<br>**[Was the readmission caused by an adverse event?]**	**Preventability**<br>**[Could the adverse event have been avoided?]**
1	No evidence for causation	No evidence for preventability
2	Slight evidence for causation	Slight evidence for preventability
3	Causation less than 50–50 but close call	Preventability less than 50–50 but close call
4	Causation more than 50–50 but close call	Preventability more than 50–50 but close call
5	Strong evidence for causation	Strong evidence for preventability
6	Virtually certain evidence for Causation	Virtually certain evidence for Preventability

Score: Please indicate your scores for causation and preventability for each of the adverse event categories below:

	Medication	Procedure	Nosocomial Infection	Diagnostic Error	Management Error	System Error	Surgical Complication	Other [Please Specify]
Causation								
Preventability								
